# Childbirth expectations questionnaire – a psychometric study with a sample from brazil

**DOI:** 10.1192/j.eurpsy.2021.1616

**Published:** 2021-08-13

**Authors:** M. Barros, M. Aguiar, A.T. Pereira, A. Macedo

**Affiliations:** 1 Instituto De Psicologia Médica, Universidade de Coimbra, Coimbra, Portugal; 2 Pos Graduação Em Psicologia Da Saúde, Universidade Federal da Bahia, Vitória da Conquista, Brazil

**Keywords:** Birth Expectations, Scales, validation, childbirth

## Abstract

**Introduction:**

The Childbirth Expectations Questionnaire (CEQ; Gupton, A., Beaton, J., Sloan, J. & Bramadat, I.; 1991) evaluates the women childbirth expectation’s with 34 items organized in four dimensions: Pain and coping; Significant others; Nursing support and Interventions.

**Objectives:**

To analyze the psychometric properties (construct validity using Confirmatory Factor Analysis, discriminant validity and reliability) of the Brazilian preliminary version of CEQ.

**Methods:**

350 women (Mean age: 30.01±5.452) in the second trimester of pregnancy (Mean weeks of gestation=25.17±6.55), with uncomplicated pregnancies, completed the CEQ. To analyze discriminant validity, thirty of these women participated in a workshop (12 hours, integrated in the GentleBirth, a specific perinatal education intervention program) and fill in the CEQ again after approximately 8 weeks.

**Results:**

After deleting seven items (1-3-20-24-33-34-35) and some errors were correlated the four-dimensional second-order model of CEQ presented good fit (χ^2^=2.496; RMSEA=.071; CFI=.845, TLI=.828). The CEQ Cronbach’s alpha for the total was α=.90; all factors presented good reliability: Pain coping (α=.87); Significant others (α=.66), Nursing support (α=.84), and Interventions (α=.76). The CEQ mean scores (total, Pain coping and Nursing support) were significantly higher after the workshop, indicating more positive expectations for childbirth (p<.05).

**Conclusions:**

This additional validation study emphasizes that CEQ is an adequate measure of expectations of labour. It will be very useful to understand the correlates of childbirth expectations and also to access the efficacy of childbirth preparation programs.

